# The Effect of Lymph Nodes' Histologic Response on Survival Outcomes in Moroccan Patients with Rectal Cancer

**DOI:** 10.1155/2020/8406045

**Published:** 2020-01-06

**Authors:** Ihsane El Otmani, Fatima El Agy, Mohammed El Abkari, Karim Ibn Majdoub Hassani, Khalid Mazaz, El Bachir Benjelloun, Khalid Ait Taleb, Touria Bouhafa, Zineb Benbrahim, Sidi Adil Ibrahimi, Laila Chbani

**Affiliations:** ^1^Laboratory of Biomedical and Translational Research. University of Medicine and Pharmacy of Fez, University Sidi Mohammed Ben Abdellah of Fez, 30070 Fez, Morocco; ^2^Laboratory of Anatomic Pathology and Molecular Pathology, University Hospital Hassan II, 30070 Fez, Morocco; ^3^Unit of Medical Genetics and Oncogenetics, University Hospital Hassan II, 30070 Fez, Morocco; ^4^Department of Gastroenterology, University Hospital Hassan II, 30070 Fez, Morocco; ^5^Department of General Surgery, University Hospital Hassan II, 30070 Fez, Morocco; ^6^Department of Radiotherapy, University Hospital Hassan II, 30070 Fez, Morocco; ^7^Department of Oncology, University Hospital Hassan II, 30070 Fez, Morocco

## Abstract

Prognosis for patients with locally advanced rectal cancer remains controversial. The purpose of this study was to elucidate possible association between therapeutic effect on lymph nodes (LNs) and patient prognosis. Overall, 149 patients with rectal cancer received preoperative radiotherapy in concomitance with chemotherapy or exclusive radiotherapy before rectal excision. Microscopic examination of formalin-fixed lymph nodes was assessed for therapeutic effect. The establishment of groups combined reaction tissue types of fibrosis, colloid, and necrosis after neoadjuvant treatment was assigned. The average age was 56.38 years, ranged between 22 and 88 years, 53% were female, and 47% were men, with a sex ratio of 1 : 12. In the present study, we noticed that after a median follow-up time of 40.67 months (0–83; SD: 21.1), overall survival was statistically significant depending on age groups. Kaplan–Meier analysis showed significant differences in the rate of patients with an age under 65 years (70.64%) versus those with an age over 85 years (36.5%) (*p* < 0.001). Also, the OS was statistically significant depending on therapeutic effect groups composed of 0TE (No Therapeutic effect), C+ (presence of only colloidal effect), F+ (presence of only fibrosis tissue), and ME+ (mixture of 2 or 3 types of therapeutic effect) group. Indeed, we observed a significantly higher OS rate in the ME + group (86%) compared with the OS rate of LNs group with no therapeutic effect (57%) (*p*=0.028). Additionally, there was a significant association between the presence of fibrosis on LNs and an extended delay of more than 8 weeks to neoadjuvant treatment completion and surgery. Our study indicates that the best patient prognosis could be predicted based on tumor presenting a best pathologic effect on lymph nodes, and that delaying surgery for more than 8 weeks to neoadjuvant treatment completion improves therapeutic response on LNs.

## 1. Introduction

Rectal cancer (RC) is one of the most common cancers in incidence and mortality in the world [1, 2]. RC presents 40% of colorectal cancer and approximately 20% of digestive cancers [3]. The standard treatment for patients with advanced rectal cancer is preoperative chemoradiotherapy or radiotherapy followed by surgery. This strategy allows to decrease the tumor size as well as to increase the degree of tumor response to neoadjuvant treatment [4]. Furthermore, the prognosis of patients with RC can be estimated based on various factors, such as tumor regression grading (TRG), vascular invasion, and perineural invasion [5, 6]. Lymph node (LN) status appears to occupy a more critical role in therapeutic strategy decisions after surgical procedure [7]. Therefore, the presence of positive lymph nodes is considered to be a poor prognosis value of metastasis and recurrence [8]. Semiquantitative evaluation of primary tumor regression on surgical specimens, after neoadjuvant treatment, was assessed by different systems [9]. The therapeutic effect on lymph nodes and primary tumor has been proposed as an indicator to select patients who may be at risk for recurrence and metastasis for patients with rectal cancer [10]. Unfortunately, none of these have been sufficiently informative for inclusion in clinical practice as recommended in breast cancer classification by several authors [11, 12]. Additionally, interval between neoadjuvant therapy completion and surgery procedure has an effect on tumor shrinkage as well as downstaging [13].

Altogether, therapeutic effect on LNs, in addition to the duration between the end of neoadjuvant therapy and surgery, could be proposed as a new challenge raised to become a part of the individualized therapy of rectal cancer indicated in clinical oncology.

Herein, we aim to describe the therapeutic effect on LNs after neoadjuvant treatment and to verify the hypothesis suggesting that prognostic value depends on therapeutic effect on lymph nodes after neoadjuvant treatment followed by surgical procedure.

## 2. Patients and Methods

### 2.1. Patients Enrollment

**Figure 1:**
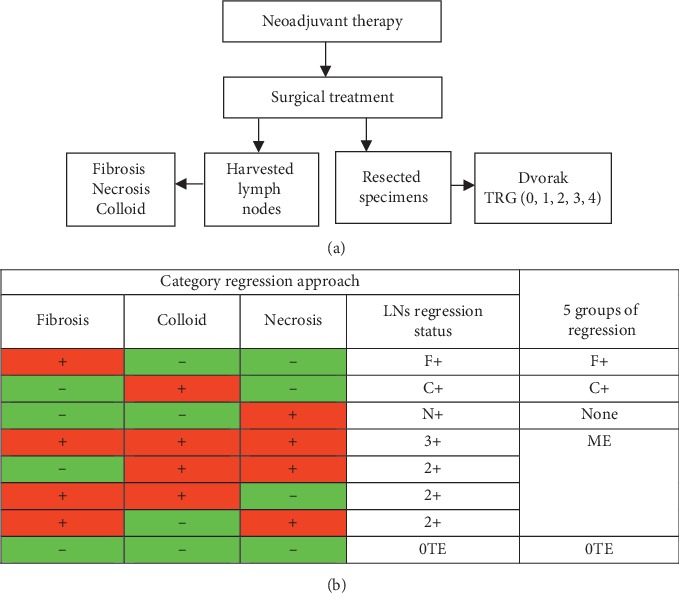
Illustration of lymph nodes regression on categorical approach. (a) Chart illustration of patient strategy enrollment. (b) Assignment of categorical regression approach, defining lymph nodes regression status. TE: therapeutic effect; LNs: lymph nodes; 0TE: absence of therapeutic effect; 2+: combination of two therapeutic effect types; 3+: combination of three therapeutic effect types; F+: fibrosis effect; C+: colloid effect; N+: necrosis effect; ME: mixed effect.

Consecutive 149 patients diagnosed for rectal cancer were enrolled in this study, at HASSAN II University Hospital Center of Fez. Patients underwent curative treatment based on proper surgery preceded by neoadjuvant exclusive radiotherapy (RT) or neoadjuvant chemoradiotherapy (CRT). Fresh specimens were transported to the department of pathology for specific procedures. Demographic and clinicopathological characteristics were collected from the pathologic database of the laboratory of anatomic pathology of HASSAN II University Hospital of Fez as well as the preoperative medical records as described in Figure 1(a).

### 2.2. Microscopic Lymph Nodes Therapeutic Effect Evaluation

Gastrointestinal pathologists assessed lymph nodes evaluation procedure. The LNs were identified by manual palpation and macroscopic examination through mesorectum cut into tight sections and then retrieved entirely and placed in separate cassettes. LNs evaluation was performed after 10% formalin fixation for a period of 48 hours, which could be extended to 72 hours if necessary.

Standard hematoxylin-eosin staining was used to assess histologic examination on microscopic sections of tissue samples and LNs. Pathologic grading was evaluated according to the TNM (tumor-node-metastasis) staging system of the 7^th^ version [14].

Presence of fibrosis with mucous substance, colloid reaction, and necrosis tissue was considered as three types of tumor response of LNs to neoadjuvant therapy. Evaluation of tumor regression was assessed by qualitative estimation of presence or absence of one or mixed reaction types (2 or 3 tumor type regression combined). F+ corresponds to the presence of only fibrosis, C+ to the presence of only colloidal tissue, N+ to the presence of only necrosis, and 2+ and 3+ correspond, respectively, to the presence of combined 2 and 3 therapeutic effect types, as described in Figure 1(b). Association of tumor regression types with overall survival and relapse-free survival was analyzed.

### 2.3. Ethic Statement

The local ethics committee at the Faculty of Medicine and Pharmacy of Fez and Hassan II University Hospital reviewed and approved the research protocol under the number 26/17. Ethical standards of the Helsinki Declaration were respected, and informed consent was obtained from all patients.

### 2.4. Follow-Up

The starting point of the calculation of OS and RFS was on 01 January 2012, and the final peak date was 30 October 2018. Overall survival (OS) was defined as the time interval from the date of diagnosis until the date of death from any cause and was censored at the last follow-up if no death was recorded. Relapse-free survival (RFS) was defined as the time between the diagnosis of the first local recurrence and distant metastasis.

### 2.5. Statistical Analysis

Effect of tumor regression on overall survival (OS) and relapse-free survival (RFS) were estimated using Kaplan–Meier curves. *p* ≤ 0.05 was considered statistically significant. For statistical analysis, SPSS 21 (Statistical Package for Social Science) was used.

## 3. Results

### 3.1. Patients' Characteristics

Patients and tumor characteristics at baseline are described in Table 1. This study consisted of 79 (52.7%) women and 70 (46.7%) men, with a sex ratio of 1.12. The majority of the population studied was young with a proportion of 68% (*n* = 102) under 65 years. Among all patients, 97.3% were classified as adenocarcinoma, and 2.7% were mucinous and signet ring cell carcinoma.

### 3.2. Therapeutic Effect Characteristics

**Figure 2:**
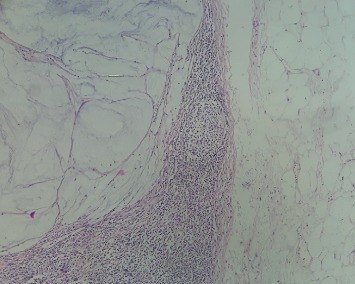
H&E staining showing a necrosis effect on lymph node after neoadjuvant treatment (magnification: ×200).

The therapeutic effect was described as the presence of fibrosis, necrosis, and colloid changes after therapeutic effect on LNs as shown in Figure 2. Among the different grouped effects, we found that fibrosis reaction was assessed in 133 cases (91.3%) associated or not with other therapeutic effect types. Colloid changes were found in 66% of cases associated or not with fibrosis and necrosis, while necrosis was associated in all cases with fibrosis and necrosis effect at a rate of 60.7% (Table 1).

### 3.3. Prognostic Value of Therapeutic Effect

**Figure 3:**
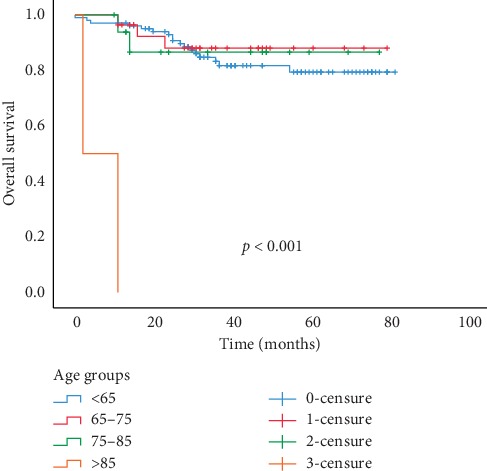
Kaplan–Meier curves for the duration in months of OS according to patient's age groups.

**Figure 4:**
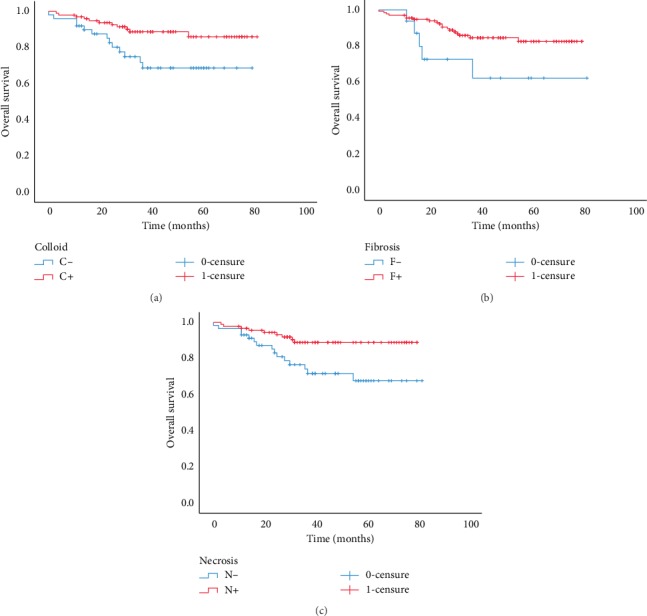
Kaplan–Meier curves for the duration in months of OS according to the (a) presence of colloid (C+: presence of colloid and C−: absence of colloid), (b) presence of fibrosis (F+: presence of fibrosis and F−: absence of fibrosis), and (c) necrosis effect (N+: presence of necrosis and N−: absence of necrosis).

The median follow-up time was 40.67 months (0–83; SD: 21.1). The OS was found to be statistically significant depending on age groups as shown in Figure 3. The OS rate of patients with an age under 65 years was 70.64% versus 36.5% of those with an age over 85 years (*p* < 0.001) (Table 2). The presence of therapeutic effect of fibrosis, colloid, and necrosis was statistically associated with prolonged OS, (*p*=0.038, *p*=0.001, and *p*=0.009), respectively, as observed in Figure 4. When grouping therapeutic effect on 4 groups of 0TE (no therapeutic effect), C+ (presence of only colloidal effect), F+ (presence of only fibrosis tissue) and ME+ (mixed of 2 or 3 types of reaction) the OS was statistically significant. Indeed, we observed a high OS rate (86%) in the ME + group compared with a low OS rate (57%) for LNs without therapeutic effect (*p*=0.028). RFS associated with different characteristics did not show statistical significance in all the analyses.

Data related to the interval between neoadjuvant therapy and surgery were available for 123 cases. The median interval between the end of neoadjuvant therapy and surgery was 8 weeks (SD: 8.1, interval: 2 to 63 weeks). 40.7% (*n* = 50) of patients underwent surgery in less than 8 weeks versus 59.3% (*n* = 73) after 8 weeks. The analysis of the effect of duration time between the end of neoadjuvant treatment and surgery on the presence of each type of responses in LNs showed a significant association between an extended delay and the presence of fibrosis. 95.9% of patients who underwent surgery after more than 8 weeks to the end of neoadjuvant treatment express fibrosis effect on LNs (*p*=0.014) as shown in Table 3.

## 4. Discussion

Neoadjuvant treatment based on exclusive radiotherapy or chemoradiotherapy followed by surgical resection is the current practice in locally advanced rectal cancer (T3 or T4 tumors, with or without lymph nodes metastasis) [15]. Lymph node status is recently the powerful indicator factor for prognosis in posttherapeutic rectal cancer [8, 16]. General guidelines recommend the evaluation of a number of 12 regional LNs to validate an accurate ypN0 status [4, 14, 15]. Unfortunately, until today, there are no particular recommendations focusing on the histologic response on retrieved LNs in rectal cancer after neoadjuvant treatment.

In this purpose, we determined a therapeutic effect in LNs, based on histological examination, likely to be a prognostic factor. Furthermore, we suggest a consideration of nodal histologic response within the staging system for rectal cancer and in the final report of pathologists.

Several grading systems have been proposed for TRG. Ryan et al. [17], Mandard et al. [18], and Dworak et al. [19] TRG grading systems are systematically and frequently used as recommended references, while these systems indicate low reproducibility and concordance in results among gastrointestinal pathologists and did not include lymph node pathologic response [20].

In our study, we found that a better overall survival was associated with the presence of combined histologic response allowed by two or all fibrosis, necrosis, and colloid compared with the group without therapeutic effects. The more the combination is large the better is the survival.

*Soo Hee Kim* has established a modified Dworak TRG system for the estimation of histologic response in combination between the primary tumor and regional lymph nodes. He compared the predictive value of different systems of TRG, as Dvorak, Rayan, AJCC, and mDworak TRG systems. Kim et al. concluded that overall survival and relapse-free survival can be better predicted using combination of ypStage and the modified Dworak TRG than using ypStage separately [10].

On the contrary, Sataloff compared overall survival between breast cancer patients groups of complete response and incomplete response. He concluded that 79% of overall survival rate was observed in patients with complete response in contrast to those showing incomplete response with a rate of 34% [12].

Regarding the effect of the interval between neoadjuvant treatment and surgery on the therapeutic response estimated posteriorly on the LNs, to date, no previous studies have assessed this research area on rectal cancer. Yet, this effect has been studied on the surgical excision specimen after neoadjuvant treatment. The study of François Y. et al. found that a short interval had less complete response effect, and 10.3% of patients who underwent surgery in week 2 had a complete response in surgical specimen versus 26% for those who had surgery between the 6th and the 8th week after radiotherapy (*p*=0.005) [21]. Additionally, Kalady et al. demonstrated in their study that a delay greater than 8 weeks was statistically correlated with the complete pathologic response [22]. In our series, we proved that patients operated after a delay greater than 8 weeks to the neoadjuvant therapy express more fibrosis therapeutic effect on LNs.

Indeed, the most consistent predictive system of prognosis and pathologic response after neoadjuvant treatment is the pathologic staging system, when sided in complete response and incomplete response.

Our investigation has accurately predicted overall survival in patients who have received primary neoadjuvant treatment for rectal cancer. This should be included in pathologist reports and taken into consideration by clinicians in the therapeutic management after surgery.

## 5. Conclusion

This study suggests that the best patient survival outcome is related to tumors presenting the best pathologic effect on nodal tissue. Moreover, pathologic response on regional LNs may be an enhancer for the prognostic value of TRG systems evaluating the primary tumor separately. Delaying surgery for more than 8 weeks to neoadjuvant treatment completion improves therapeutic response on lymph nodes.

## Figures and Tables

**Table 1:** Patient and therapeutic effect on lymph nodes characteristics.

	Number of patients (%)
Age	
65	102 (68.45)
65–75	28 (18.8)
75–85	17 (11.4)
85	2 (1.35)

Sex	
Men	70 (46.97)
Women	79 (53.03)

Histologic type	
Adenocarcinoma	145 (97.3)
Mucinous/signet ring cell	4 (2.7)

Degree of differentiation	
Well	72 (48.3)
Moderate	69 (46.3)
Poor	8 (5.4)

Therapeutic response on surgical specimen	
Complete response	25 (16.8)
Incomplete response	124 (83.2)

Therapeutic effect on surgical specimen according to the percentage of tumor regression	
Presence	137 (92)
Absence	12 (8)

Fibrosis TE on LNs	
Presence	133 (89.3)
Absence	16 (10.7)

Colloid TE on LNs	
Presence	99 (66.5)
Absence	50 (33.5)

Necrosis TE on LNs	
Presence	91 (61.07)
Absence	58 (38.93)

Regression types grouped on 5	
0TE	12 (8.1)
2+	8 (5.4)
3+	89 (59.8)
F+	36 (24)
C+	4 (2.7)

Regression types grouped on 4	
0TE	12 (8.1)
F+	36 (24.2)
C+	4 (2.7)
ME+	97 (65)

TE: therapeutic effect; LNs: lymph nodes; 0TE: absence of therapeutic effect; 2+: combination of two therapeutic effect types; 3+: combination of three therapeutic types; F+: fibrosis effect; C+: colloid effect.

**Table 2:** Univariate analysis for OS and RFS among patients with rectal cancer according to different characteristics.

Characteristics	Overall survival			Relapse-free survival		
	OS (%)	95% CI	*p*	RFS (%)	95% CI	*p*
Age						
65	70.64	65.74–75.55	<0.001	NA	—	0.427
65–75	72.46	64.43–80.49		NA	—	
75–85	69.19	57.80–80.58		NA	—	
85	36.50	0.00–15.32		NA	—	

Sex						
Men	69.75	63.49–76.01	0.600	59.46	50.99–67.92	0.684
Women	69.53	64.10–74.96		60.18	52.54–67.81	

Histologic type						
Adenocarcinoma	70.71	66.50–74.91	0.607	NA	—	
Mucinous/signet ring cell	57.00	24.74–89.25		NA	—	

Therapeutic effect						
Absence	47.31	33.53–61.09	0.041	36.21	18.53–53.89	0.098
Presence	71.69	67.52–75.85		61.74	55.82–67.88	

Fibrosis TE on LNs						
Absence	58.84	42.45–75.23	0.038	49.57	27.70–71.44	0.300
Presence	70.27	66.23–74.31		60.06	54.25–65.87	

Colloid TE on LNs						
Absence	61.69	53.27–70.10	0.001	54.66	44.23–65.10	0.279
Presence	74.15	69.79–78.51		62.74	55.98–69.50	

Necrosis TE on LNs						
Absence	63.62	55.74–71.50	0.009	58.42	48.67–68.17	0.549
Presence	73.26	69.07–77.44		60.30	53.33–67.26	

Regression types grouped on 5						0.427
0TE	47.31	33.53–61.09	0.051			
2+	71.80	64.43–79.16		NA	—	
3+	73.11	68.84–77.38		NA	—	
C+	60.00	24.79–95.20		NA	—	
F+	62.81	53.19–72.43		NA	—	

Regression types grouped on 4						0.331
0TE	47.31	33.53–61.09	0.028	NA	—	
C+	60.00	24.79–95.20		NA	—	
F+	62.81	53.19–72.43		NA	—	
ME+	72.98	68.86–77.09		NA	—	

TE: therapeutic effect; LNs: lymph nodes; 0TE: absence of therapeutic effect; 2+: combination of two therapeutic effect types; 3+: combination of three therapeutic types; F+: fibrosis effect; C+: colloid effect; NA: not applicable.

**Table 3:** The effect of neoadjuvant to surgery interval on the therapeutic effect type.

Therapeutic effect	Delay between the end of neoadjuvant treatment and surgery		*p* value
	<8 weeks	>8 weeks	
Fibrosis			0.014
Absence	9 (18%)	3 (4.1)	
Presence	41 (82%)	70 (95.9%)	

Necrosis			0.259
Absence	23 (46%)	25 (34.2%)	
Presence	27 (54%)	48 (65%)	

Colloid			0.703
Absence	16 (32%)	26 (35.6%)	
Presence	34 (68%)	47 (64.4%)	

## Data Availability

The data used to support the findings of this study are available from the corresponding author upon request.
